# An integrative approach to understanding microbial diversity: from intracellular mechanisms to community structure

**DOI:** 10.1111/j.1461-0248.2010.01507.x

**Published:** 2010-09

**Authors:** Ivana Gudelj, Joshua S Weitz, Tom Ferenci, M Claire Horner-Devine, Christopher J Marx, Justin R Meyer, Samantha E Forde

**Affiliations:** 1 Department of Mathematics, Imperial College LondonLondon SW72A7, UK; 2 School of Biology, Georgia Institute of Technology and School of Physics, Georgia Institute of TechnologyAtlanta, GA 30332, USA; 3 School of Molecular and Microbial Biosciences, University of SydneySydney 2006, Australia; 4 School of Aquatic and Fishery Sciences, University of WashingtonWashington, DC 98105, USA; 5 Department of Organismic and Evolutionary Biology, Harvard UniversityCambridge, MA 02138, USA; 6 Zoology Department, Michigan State UniversityMI 48824, USA; 7 Ecology and Evolutionary Biology Department, 1156 High St., University of CaliforniaSanta Cruz, CA 95064, USA

**Keywords:** Ecological genomics, experimental evolution, mathematical models, micro-organisms, metabolism, parasitism, trade-offs, viruses

## Abstract

Trade-offs have been put forward as essential to the generation and maintenance of diversity. However, variation in trade-offs is often determined at the molecular level, outside the scope of conventional ecological inquiry. In this study, we propose that understanding the intracellular basis for trade-offs in microbial systems can aid in predicting and interpreting patterns of diversity. First, we show how laboratory experiments and mathematical models have unveiled the hidden intracellular mechanisms underlying trade-offs key to microbial diversity: (i) metabolic and regulatory trade-offs in bacteria and yeast; (ii) life-history trade-offs in bacterial viruses. Next, we examine recent studies of marine microbes that have taken steps toward reconciling the molecular and the ecological views of trade-offs, despite the challenges in doing so in natural settings. Finally, we suggest avenues for research where mathematical modelling, experiments and studies of natural microbial communities provide a unique opportunity to integrate studies of diversity across multiple scales.

## Introduction

Trade-offs are ubiquitous at all scales of life. For instance, whether between colonization and competition, virulence and transmissibility, or nutrient uptake and cell division, trade-offs are often invoked as explanations for how and why species diversify and coexist. Kneitel & Chase (2004) advocate that a ‘better understanding of the range, variation and interactions of trade-offs at multiple spatial scales’ is key to the development of a more synthetic view of diversity. However, the molecular basis of trade-offs is often treated as a black box (Kneitel & Chase 2004; Litchman & Klausmeier 2008; Angert *et al.* 2009). This is unfortunate because trade-offs may remain misidentified, overlooked or insufficiently characterized in the absence of mechanistic studies. Instead, in this study, we argue that integrating molecular understanding of trade-offs within an ecological context provides the best hope for predicting if and how trade-offs emerge from intracellular processes, if and how they evolve, and what their impact is on diversity.

We focus on microbes as a means to connect the intracellular basis of trade-offs to their ecological impacts. There are a number of reasons for exploring this approach in the context of microorganisms. First, a better understanding of trade-offs will help resolve unanswered questions about the organization and functioning of microbial communities, such as the relationship between microbial diversity and habitat area (Horner-Devine *et al.* 2004; Prosser *et al.* 2007) and the relationship between microbial diversity and productivity (Kassen *et al.* 2000; Horner-Devine *et al.* 2003). Next, microbes are unusually amenable to studies that unite intracellular mechanisms with ecology and evolution because: (i) eco-evolutionary manipulations of microbes are readily available and (ii) regulatory and biophysical studies with microbes can provide data to study sequences, structure and function as well as develop genotype to phenotype maps (Ptashne & Gann 2002; Alon 2006). Finally, unlike higher taxa, much of the microbial world remains undiscovered. Only 1–10% of microbes are culturable and we have just recently been able to identify many species with the advent of molecular-based sequence diversity approaches. However, a significant challenge remains: what is the ecological and physiological relevance of this sequence diversity? We make a case that a combination of mathematical models, microbial experiments in the laboratory, and *in situ* detection of diversity is a next-generation synthesis that will allow rapid advances in our understanding of mechanisms and patterns of microbial and viral diversity.

We begin by examining how constraints at the intracellular level lead to the emergence of trade-offs at the ecological level focusing primarily on two examples: (i) metabolic and regulatory trade-offs in bacteria and yeast and (ii) life-history trade-offs in bacterial viruses. Analysis of these two trade-off examples focuses on experimental systems and model organisms well suited to mathematical modelling. Next, we highlight ongoing efforts to understand the intracellular basis for trade-offs and its impact on diversity of marine microbes such as phytoplankton, cyanobacteria and cyanophages. Although making the jump from laboratory to natural populations is challenging, we describe evidence for successes that have already taken place and cautions regarding when trade-offs do not exist or cannot be identified. We note that in many cases, steps have been taken to link mechanisms across scales to understand patterns of diversity as we propose here, however, there are few examples that do so in entirety. As such, we end with a discussion of prospects for continued discoveries.

## Metabolic and regulatory trade-offs within microbes

**Figure 1:**
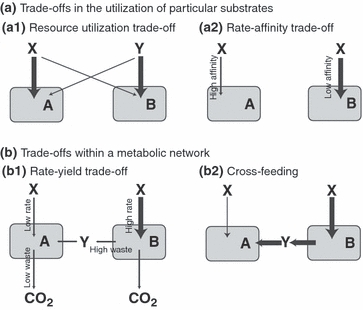
Trade-offs in the (a) utilization of particular substrates and (b) metabolic strategies that drive bacterial diversity with thick arrows representing fast rates and thin lines representing slow rates of resource utilization or excretion: (a1) resource utilization trade-off where type A is best adapted to resource X whereas type B is best adapted to resource Y; (a2) rate-affinity trade-off where bacteria can adapt alternative transport pathways to resource utilization: type A is transporting resource X slowly through a high-affinity pathway and type B is transporting the same resource quickly through a low-affinity pathway as observed in Muir *et al.* (1985) and Maharjan *et al.* (2006). (newb1) Rate yield trade-off whereby type A converts resource X into energy slowly but efficiently while type B utilizes the same resource X quickly but inefficiently and as a consequence excretes a metabolite Y that can be used as an additional energy source by type A; (newb2) Cross-feeding where type B is adapted to utilize the primary resource X but in the process excretes a metabolite Y that is then utilized by type A as discussed in Rozen & Lenski (2000), Friesen *et al.* (2004) and Porcher *et al.* (2001).

In all parts of the biosphere microorganisms can be found with an immense range of metabolic capabilities and the remarkable capacity to survive profound environmental stresses. Given the enviable metabolic and regulatory flexibility possessed by many microbes, one might imagine a world with just one or a few superbugs that can eat everything and survive anything that comes their way. As it turns out, it would be hard to conceive of a scenario further from the truth, as nearly all natural communities are comprised of myriad species with distinct metabolic and survival strategies; e.g., diversity is estimated to be on the order of 10^4^ species per cubic centimetre of soil (Torsvik *et al.* 2002). The trade-off costs in specializing on particular lifestyles are thought to contribute to the evolution of diverse fitness solutions. In this section, we highlight three examples to illustrate that the marriage of mechanistic studies of trade-offs with mathematical models can shed light upon how ecological outcomes arise as the consequence of metabolic and regulatory capacities of the individual genotypes in a community. There are of course many other studies of trade-offs (e.g. Muir *et al.* 1985; Treves *et al.* 1998; Rozen & Lenski 2000; Porcher *et al.* 2001; Friesen *et al.* 2004; Maharjan *et al.* 2006; Spencer *et al.* 2007; see also Fig. 1a2, b2) but we do not discuss them here because in these cases, the links between mechanistic studies, mathematics and ecology have not been fully integrated. The examples we have chosen highlight sources of divergence in the utilization of particular substrates, metabolic strategies and regulatory networks.

### Trade-offs in the utilization of particular substrates: how to eat at a buffet

**Figure 2:**
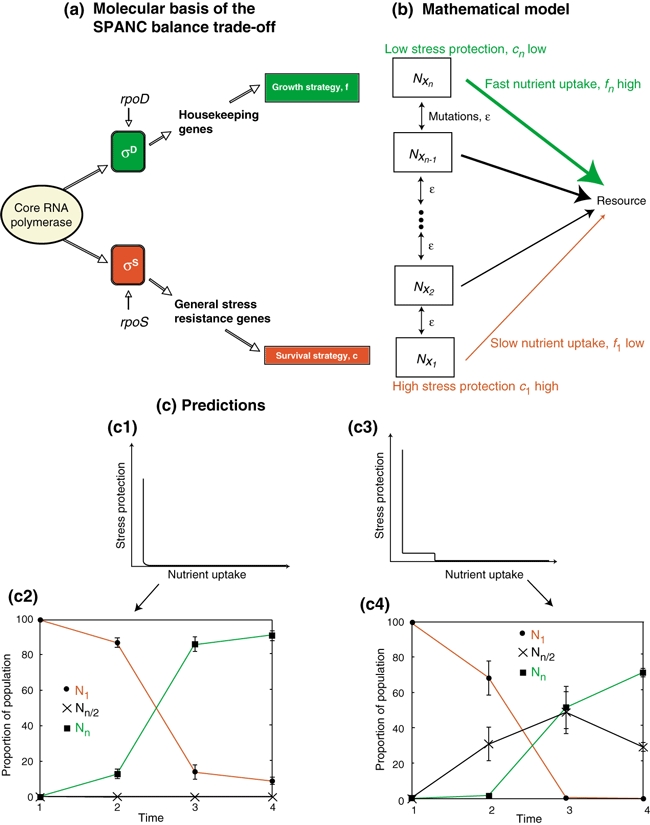
(a) Molecular basis of the SPANC balance trade-off. (b) A schematic of a mathematical model incorporating the SPANC balance trade-off. The model considers an *E. coli* population with *n* competing strains each with a different value of the *rpoS* expression *x* so that 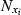
is the density of a strain with phenotype *x*_*i*_ where *i = 1…n* and *0*= *x*_1_*≤ x*_2_*≤…≤ x*_*n*_*= 1*. Evolutionary changes are constrained by the SPANC balance trade-off in the following way: an increase in *rpoS* expression (*x*_*i*_) leads to a decrease in nutrient uptake (*f*_*i*_) and an increase in stress protection (*c*_*i*_)*.* Bacterial growth is proportional to the rate of ATP production while mutations altering *x* occur at a rate *ε.* The model predicts that the SPANC balance trade-off shape illustrated in (c1) could give rise to the experimentally observed mutational sweeps for temperature stress of 44°C shown in (c2) whereas the SPANC balance trade-off shape illustrated in (c3, see also King *et al.* 2006) could give rise to the experimentally observed mutational sweeps for the acid stress of pH 6.0 illustrated in (c4, see also King *et al.* 2006). The model also predicts that trade-off shape influences the long-term variation in stress protection (i.e. the *rpoS* expression). These results provide an insight into possible mechanisms governing the evolution of diverse stress-responses in bacteria.

A trade-off between specificity toward one substrate vs. another (Fig. 2a1) could arise as a consequence of the fact that enzymes have multiple phenotypes for which mutations can have opposite effects. Lunzer *et al.* (2002) considered two *Escherichia coli* strains that differed only with respect to possessing distinct lactose utilization operons that encoded lactose permeases (product of *lacY*) with differing, alternate specificities for methyl-galactoside and lactulose (Silva & Dykhuizen 1993; Dean 1995). The potential for coexistence was determined by the fitness of each strain when either rare or dominant in the population. For a range of concentrations of the two galactosides, *either* strain could invade when rare, the hallmark of negative-frequency dependent coexistence. Lunzer *et al.* (2002) then generated a quantitative prediction of steady-state community composition via a simple model of growth for each strain using a series of linked equations based upon metabolic control analysis (Kascer & Burns 1973; Heinrich & Rapoport 1974a,b;). The key assumption was that the predicted rate of flux through the initial reactions for either galactoside set the pace of growth in their experiments. The computational analysis recapitulated both the qualitative phenomenon of coexistence over an intermediate range of galactoside concentration ratios as well as generated a remarkably accurate prediction of just how narrow this window should be (*c.*72–80% lactulose). These results indicate that in an environment containing two limiting resources, coexistence of two competitors each specializing on a different resource is possible only within a narrow range of resource ratios. However, a subsequent study tracking the fate of this balanced polymorphism over evolutionary time suggested that, once stabilized, ecological specialization prevented selective sweeps through the entire population thereby promoting the coexistence of the two ‘buffet’ specialists (Dykhuizen & Dean 2004).

### Trade-offs within metabolic networks: haste makes waste

Trade-offs between the rate of substrate use and the yield of biomass generated illustrate how integrating physiological mechanisms and mathematics can explain ecological coexistence of microbial strains with alternative ways of metabolizing the same resource. From the perspective of thermodynamics and kinetics, rate and yield ultimately must come at odds with one another at some limit: as the harvest of free energy from a reaction gets closer to 100%, it saps the driving force for the transformation and moves the system toward equilibrium (i.e. zero net flux). This trade-off has been shown for glucose utilization in yeast to be sufficient to permit coexistence in seasonal environments (MacLean & Gudelj 2006). In this case, the high rate but low yield strategy can be stably maintained with the low rate but high yield strategy. A mathematical model of simplified biochemistry (a cell being depicted as just three conglomerate reactions, Fig. 1b1), under the assumptions that (i) the rate of ATP generation could approximate fitness and (ii) accumulation of the one metabolic intermediate would be toxic, led to quantitative predictions that were in accord with the empirical data. Interestingly, rather than being imposed and fixed, the rate-yield trade-off varied with the environment and was an emergent property of the model. However, rate-yield trade-offs have not been observed in all cases. Examination of the rate and yield of *E. coli* evolved for 20 000 generations in glucose medium found that these two factors were positively correlated overall (Novak *et al.* 2006). The lack of an observed trade-off may indicate that these biochemical pathways may have operated sufficiently far from thermodynamic constraints that improvement in both dimensions was possible, at least initially.

### Trade-offs within regulatory networks: microbial stress management skills

Microbes encounter highly heterogeneous stresses in addition to heterogeneous nutrients. Environmental variables such as temperature and acidity are known to have a pervasive effect on bacterial growth (Ingraham 1987). For example, in *E. coli*, resistance to high temperatures is crucial for surviving sanitizing treatments of food products (Yuk & Marshall 2003) whereas acid resistance is essential for survival in the gastrointestinal tract (Foster 2004). It should come as no surprise that transcriptional regulatory networks that underlie stress management and nutrient uptake are diverse within and between bacterial species (Lozada-Chávez *et al.* 2006). In handling stress and nutrients, there is a potential for a trade-off where resource uptake and stress resistance may be negatively correlated. This trade-off is often seen in species ranging from plants (Sabreen & Sugiyama 2008) to *Drosophila* (Hoffmann *et al.* 2001) to *E. coli* (Ferenci & Spira 2007) where it was termed a *s*tress *p*rotection *a*nd *n*utritional *c*apability (SPANC) balance trade-off (Ferenci 2005).

The molecular basis of the SPANC balance trade-off is well understood in *E. coli*. The stress resistance involves expression of several hundred genes belonging to the general stress response, which is triggered by environmental stresses, starvation and reduced growth rate. The response is co-regulated by a global transcriptional regulator called RpoS or σ^S^, a sigma factor that directs RNA polymerase to genes involved in stress resistance (Hengge-Aronis 2002). As it turns out, the major cost of stress resistance is not the cost of transcribing additional genes, but the fact that elevated RpoS levels lead to decreased expression from the sigma factor RpoD (σ^D^) promoters responsible for the expression of housekeeping genes involved in most cellular functions. Because there is a finite transcriptionally available pool of RNA polymerase (Ishihama 2000), RpoS diverts transcription away from vegetative growth genes by competing with RpoD for RNA polymerase. This is the molecular cost of stress resistance in *E. coli*. The ability to metabolize and grow on most substrates is greatly reduced by σ^S^ because vegetative genes are less well expressed when σ^S^ is displacing σ^D^ from RNA polymerase (King *et al.* 2004). Variation in SPANC settings among natural isolates of *E. coli* is due to distinct levels of σ^S^, which results in organisms that are more or less stress-resistant and nutritionally competent in the same environment (King *et al.* 2004).

The cause of the difference in σ^S^ levels in different strains is either polymorphism in *rpoS* itself (Ferenci 2003), or in the regulation of RpoS levels by the processing of various stress signals. Resetting of the SPANC balance in the direction of decreased stress resistance but better nutritional capability is often through partial or complete inactivation of *rpoS*, and such *rpoS* mutant strains are found amongst natural isolates. Furthermore, there is plenty of genetic scope for nutritional- or stress-related selective pressures working in opposite directions (antagonistic pleiotropy) to result in polymorphisms affecting σ^S^ (Fontaine *et al.* 2008; Ferenci *et al.* 2009). Regulation provides a limited fine control for maintaining the right level of adaptation in a particular niche but mutational changes provide the coarse control for adaptation between the species-wide environments of *E. coli,* hence broadening the capabilities of a species.

By using mathematical models, many basic questions regarding evolution of diversity may be solvable once the molecular basis of trade-offs is understood. For example, in a recent study by S. Nilsson, T. Ferenci, K. Haynes & I. Gudelj (unpublished data), a mathematical model was developed to test whether the SPANC balance trade-off (Fig. 2a) could be a major cause of observed heterogeneities in the bacterial core stress response gene *rpoS*. The model was built on a series of simplifying molecular and metabolic cell level assumptions that were subsequently incorporated into the population level framework (Fig. 2b). This allowed the authors to tease apart the effect of the SPANC trade-off on the evolution of stress protection in microorganisms. Consistent with the experimental data the model indicates that different environmental stresses alter the molecular underpinnings of the SPANC balance trade-off changing its shape, which in turn influenced the mutational sweeps and the long-term diversity of the microbial population (Fig. 2c). This example of microbial stress management in the form of a global transcriptional regulator RpoS, indicates that it is possible to use molecular mechanisms as a solid basis for analyses of trade-offs as well as for the generation of testable theoretical predictions regarding maintenance of diverse stress responses in microbes.

## Molecular trade-offs within bacterial viruses

Trade-offs that arise in the parasitic exploitation of hosts reflect a challenge similar to that in metabolic trade-offs among bacteria: how best to utilize resources given biophysical constraints. For bacteriophages (viruses that attack bacteria; or phages), exploitation of host resources support an inter- and an intracellular life history phase. Outside of hosts, phages passively diffuse. Upon adsorption with a susceptible host, phages inject their genetic material and utilize cellular machinery to either lyse the cell thereby releasing new virions, or enter lysogeny, where the phage integrates its genetic material with the host leading to a latent state (Weinbauer 2004). We will denote this initial bifurcation in cell fate as the lysis-latency decision, although it is not the only possible outcome of a phage infection.

When the lytic pathway is initiated, the release of virions depends on a cascade of timed events. Both the time between lytic initiation and lysis (the latent period) and the number of virions produced (the burst size) are highly variable and linked. Early lysis may diminish the probability of alternative cell fates (e.g. the cell is eaten by a protist or infected and killed by another virus) and later lysis may provide more time for increased phage production – this is a form of a rate-yield trade-off as described in the previous section. Once outside the cell, virions are unstable and their de-activation rate is highly variable depending on both genotype and environmental conditions, with lifetimes ranging from hours to days.

Nearly, all functional traits of phages exhibit multiple orders of magnitude of variation (Table 1). In addition, some viral traits are highly correlated, for example there exists a strong positive correlation between the extracellular de-activation rate of viruses and the intracellular multiplication rate of viruses (De Paepe & Taddei 2006). This trade-off is similar to classic quality vs. quantity trade-offs found in reproduction in macro-organisms. For other traits, the functional forms of adaptive traits are constrained in ways that may not be expected without accounting for the intracellular mechanisms that give rise to the trait in the first place. To illustrate this point, we next describe ongoing efforts to link detailed molecular studies of viral gene regulation to the lysis-latency decision.

**Table 1:** Functional trait diversity of bacteriophages (adapted from De Paepe & Taddei (2006) with additional data on cyanophages (Sullivan *et al.* 2005). For expanded definitions of parameters see main text. In nearly all cases, there is multiple orders of magnitude of variation in trait values

Functional trait	Variable	Estimated range
Lysogny probability	ρ	0–1
Induction rate	η	10^−9^ to 10^−3^
Adsorption rate	ϕ	≈10^−8^ cm s^−2^
Half-life	1/*m*	Few hours to many days
Latent period	τ	< 1 h to many days
Burst size	β	50–3500

### Choosing between horizontal and vertical transmission: scaling up from gene regulation to a life history trait

Among temperate phages, the probability of latency upon infection denotes the relative allocation to vertical (latency) vs. horizontal (lysis) transmission. That the decision is either-or means that phages are faced with a trade-off between transmitting vertically (i.e. the genome is still integrated into the host cell when it divides), or horizontally, either of which may be evolutionarily favourable depending on ecological conditions. For the model system used to analyse temperate phages, phage λ, the probability of initiating the latent pathway is not a constant. As was first proposed over 30 years ago (Kourilsky 1973), the probability of latency depends strongly on the number of phages co-infecting a host. The same study suggested that lysogeny required a critical threshold of phages for initiation, and that the threshold differed for different phages – a suggestion that is now being re-addressed using molecular and mathematical techniques.

The molecular biology involved in determining the lysis-latency decision involves a cast of molecular players and interactions (reviewed in Ptashne 2004). The current consensus is that the decision is determined by the activity level of a single protein, CII – a regulatory protein associated with latency. When CII is high, latency ensues and when CII is low, lysis ensues. Quantitative analysis of gene expression patterns of cells that reached different fates suggested that a functional threshold of CII must be reached to establish latency (Kobiler *et al.* 2005). However, CII is just part of the complex gene regulatory network of phage λ. To understand the interaction between co-infection and cell fate, we must address how viral gene expression dynamics change when multiple phages of the same strain co-infect a cell.

**Figure 3:**
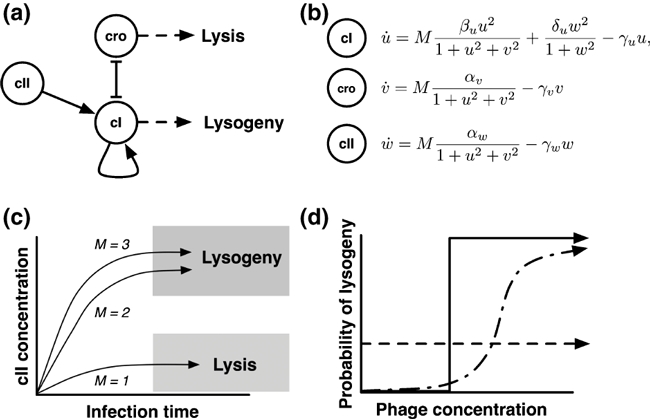
Scaling up from gene regulatory networks to intracellular concentration dynamics to phage life history traits. (a) The latency trait of phage λ is determined by the dynamics of a gating gene, *cII*, along with other genes including *cI* and *cro* (for more details see Ptashne 2004). (b) The dynamics of viral proteins can be modelled (dots denote time derivatives) as a function of protein concentrations *u*, *v* and *w*, kinetic parameters (*β*, *α* and *δ* denote maximum production rates, *γ* denotes degradation rates and subscripts denote the protein type), and the number of co-infecting phages, *M* (for detailed explanations of equations see Weitz *et al.* 2008). (c) Changing the number of co-infecting phages modifies the concentration dynamics of cII, which leads to downstream decisions, i.e. either latency or lysis, although not strictly deterministically. (d) Three possible scenarios of the key phage trait – the probability of latency – as a function of the ratio of co-infecting phages, *M*, to cell volume, a constraint suggested by recent studies (St. Pierre & Endy 2008; Weitz *et al.* 2008). For phage λ, the probability of latency is thought to be an increasing function of intracellular phage concentration. The exact dependency depends on intracellular kinetic parameters which differ among phage strains and host cell physiology.

Increasing the number of co-infecting phages will initially increase the transcription and translation rates of viral proteins. However, because of feedbacks in gene regulation, transient dynamics as well as steady-state values of viral proteins need not scale linearly with viral infection load. Instead, in a set of analyses of nonlinear models of viral gene regulation, it was demonstrated mathematically that gene expression can be *nonlinearly* related to the copy number concentration of genes (or in this case, entire viral genomes) (Mileyko *et al.* 2008; Weitz *et al.* 2008). In the case of phage λ infection, multiple infections by the same viral strain increase the number of copies of all genes of that strain. This change in viral chromosome number may drive CII expression levels above a threshold such that it turns on the latency pathway while indirectly suppressing the lytic pathway (see Fig. 3). The probability of latency is, in fact, a function of phage multiplicity of infection and host physiology.

Theoretical analysis of viral gene regulation suggests that it is not the viral genome number but rather the viral genome concentration that strongly influences cell fate determination (Weitz *et al.* 2008). Hence, for a fixed number of co-infecting viruses, theory predicts that cell fate should also depend on cell size. For example, if a small cell is infected by a single virus, the cell fate should be equivalent to two phages infecting a cell twice that size. By infecting hosts with phages at sufficiently low numbers so that hosts were always singly infected, St. Pierre & Endy (2008) were able to show that small cells tend to be fated for latency and large cells fated to lysis. Still unresolved is whether cell size is the primary driver of this effect, or is actually a proxy for some other physiological change in host state and what other stochastic factors may influence cell fate. Nonetheless, there is strong evidence that the combined effects of the multiplicity of infection, cell volume and stochasticity determine the lysis-latency decision (Zeng *et al.* 2010). Hence, the evolution of the lysis-latency decision trait in response to co-infection and cell size will be constrained. Eco-evolutionary models that aim to predict the functional diversity of these two traits via a trade-off model would be hard-pressed to have posited such a dependence in the absence of a regulatory model.

### Linking scales: from molecular mechanisms to eco-evolutionary dynamics

Although the study of the lysis vs. latency decision illustrates the incorporation of subcellular details as a means to predict trade-offs, this is just one of many possible phage traits to examine. Determining which host a phage infects, and at what efficiency it does so, is another example of a trade-off that can drive the diversity of both the phage and the host populations.

There are by now a number of models of host–phage population dynamics, for example, Levin *et al.* (1977) and Abedon (2008). These population dynamic models generally assume (i) that phages act like predators (or as parasitoids; Weitz & Dushoff 2008) with bacteria as their prey (or host); and (ii) bacterial hosts have some type of resource dependence in their reproduction. The traits that underlie such ecological models are presumed to evolve given trade-offs. In a few cases, explicit eco-evolutionary modelling has been used to predict how phage traits can evolve in simple environments, for instance, the evolution of phage latent period due to rate-yield trade-offs (Wang *et al.* 1996).

How can population dynamic models be used to predict the emergence and maintenance of large numbers of coexisting types? The diversity of phages and hosts has been shown to be intimately linked to the mechanisms that govern which phages can infect which hosts. The pattern of infection between a set of phages and a set of hosts is commonly referred to as ‘host-range structure’. Host-range structure is characterized as the pattern of cross-infectivity between a set of phages and a set of hosts (see Sullivan *et al.* 2006; Poullain *et al.* 2008). Diversifying coevolution of multiple hosts and phages on a single resource has been shown theoretically if phages can infect multiple types of hosts and when hosts suffer a penalty in growth while evolving receptor traits that diminish phage infection (Weitz *et al.* 2005). This type of diversification via coevolution depends strongly on the host-range structure of host mutants and phage mutants. A combined theoretical and empirical study demonstrated that the relationship between resource input and diversity depended strongly on the molecular details underlying phage and host trade-offs that drive host-range structure (Forde *et al.* 2008). Thus, mathematical models can help predict when detailed regulatory and biophysical mechanisms are necessary for determining the relationship between trade-offs in host range and resistance, and phage diversification. Furthermore, models can also help to predict which relationships are likely to transcend molecular details and which may vary on a case by case basis.

## From the desktop and benchtop to natural microbial communities

Thus far, we have argued that an improved understanding of diversity must include an understanding of biophysical constraints that create ecological trade-offs. However, once we move from the desktop and benchtop to natural communities, even less is known about links among ecological and subcellular scales and how trade-offs evolve and influence patterns of diversity. We discuss three examples in which there is a growing understanding of diversity across multiple biological scales of organization, and for which it is possible to link a biophysical mechanism for trade-offs with the effects of these trade-offs on patterns of diversity in the field.

### Niche differentiation with the dominant ocean autotrophs

Understanding the global distribution of ocean autotrophs would seem to be a nearly insurmountable problem, requiring global circulation models to be coupled to ecosystem models. Follows *et al.* (2007) recently attempted such a synthesis by seeding distinct phytoplankton types in a global circulation model with explicit accounting of inorganic and organic nutrient pools. These types varied based on size, trophic position, and physiological responses to light and nutrients. During simulations of this global ecosystem model, many initial types went extinct, whereas others maintained significant population densities that varied with latitiude, longitude and depth. The model phytoplankton types that persisted exhibited many similarities to observed phytoplankton including analogs of diatoms, eukaryotic phytoplankton, *Prochlorococcus*– a ubiquitous photoautotroph and other photo-autotrophs. For example, *Procholoroccus* analogues dominated in the tropical and subtropical regions – in agreement with empirical observations. However, results of this study were heavily influenced by the imposition of trade-offs that restricted the possible types of phytoplankton in the simulation (e.g. by eliminating the possibility of super-autotrophs). Litchman *et al.* (2007) further investigated the molecular basis of phytoplankton trade-offs, and found a significant negative correlation between competitive ability and reproduction using a model parameterized with data from laboratory experiments. The researchers hypothesized that this trade-off occurs as a constraint created by scaling laws of cell size: small cells reproduce faster; however, they are slower at consuming nutrients. Therefore, by explicitly incorporating data on intracellular trade-offs into their size-dependent physiological model, Litchman and coworkers were able to predict which species should dominate nutrient-rich vs. nutrient-poor environments.

### Fitness cost of viral resistance

There exists ample evidence from laboratory studies of culturable bacteria (*Escherichia*, *Salmonella* and *Pseudomonas*) demonstrating that bacterial strains can evolve resistance to viral infection and that there are associated fitness costs of resistance (Bohannan & Lenski 2000; Buckling & Rainey 2002; Harcombe & Bull 2005). However, we know much less about how and if these trade-offs affect natural communities of microbes. Lennon *et al.* (2007) set out to estimate the cost of resistance in *Synechococcus* (a marine cyanobacteria) isolated off the coast of Rhode Island, USA. The researchers measured trade-offs between resistance to multiple, naturally co-occurring viruses and fitness, demonstrating the fitness costs often documented in laboratory strains are also likely key in structuring marine microbial communities. It is thought that the fitness cost to resistance derives from a trade-off between accessibility of receptor sites on cell surfaces to phages vs. the utilization of cell surface receptors in nutrient uptake (Menge & Weitz 2009). However, the detailed biophysical and gene regulatory basis for such costs to resistance are yet to be fully mapped out, particularly because there are many ecological settings in which a resistant host has nearly identical growth rates as susceptible competitors. The environmental dependence of trade-offs in fitness costs of resistance in the study, as well as in many others, remains an open question (Bohannan & Jessup 2008).

### Intracellular exploitation of hosts by cyanophages

Latent periods of phages (the time between infection to lysis) are correlated to the doubling time of their bacterial hosts (Proctor & Fuhrman 1990). Hence, for rapidly dividing bacteria, the latent period of phages can be on the order of an hour or less. However, *Prochlorococcus*, the numerically dominant photoautotrophs in the ocean, have comparatively slow doubling times (on the order of 1–10 days). Given the additional physiological burden of phage infections in nutrient-poor waters, phages face a potential trade-off between bursting quickly with few progeny or taking a longer time to burst, but with more progeny (Wang *et al.* 1996). Recent analysis of cyanobacteria genomes have revealed that some *Prochlorococcus* strains have photosynthetic genes that modify intracellular carbon fixation during the phage lysis cycle, apparently as a means to adapt to low-nutrient environments (Sullivan *et al.* 2005). Previous notions of how a phage modifies its host-range have focused on modification to tail fibre proteins which would allow a phage to bind to previously inaccessible hosts. The finding of phage-encoded photosynthetic genes suggests that viruses may evolve to alter their position along an ecological trade-off between fecundity and the latent period, which allows them to persist on a host living in a resource-poor environment. The shape of the trade-off may also be modified by these photosynthetic genes, increasing progeny across all latent periods on average, while still retaining the trade-off between progeny number and latent period (Sullivan *et al.* 2006). Note that the same set of genes that might benefit a phage in low-nutrient environments while infecting cyanobacteria may prove detrimental in infecting bacteria in nutrient rich environments. In such cases, metabolic conversion of pre-existing bacterial carbon and nutrient sources could present a more efficient route to phage reproduction and eventual lysis. Together, these studies demonstrate that detailed molecular studies of intracellular exploitation by viruses may explain the evolution of trade-off positions and of the trade-off shape itself.

## Future directions

There has been an increasing call for the utilization of a trait-based perspective in the study of microbial ecology (Litchman *et al.* 2007; Green *et al.* 2008). However, to model microbes and their traits, it is essential to understand trade-offs among traits. As we have argued here, generalities in the shape of trait trade-offs, if they in fact exist, will stem from detailed examination of intracellular mechanisms. Such an examination will lead to advances in addressing three important questions. First, do trade-offs exist, and when they do exist, what explains the variation in their shape, magnitude and dimensionality? Second, can mechanistic analyses of trade-offs lead to a more general understanding of how trade-offs evolve (Roth & Fairbain 2007)? Explicitly addressing the variation in trade-offs can provide key insights into how and when trade-offs evolve (Roth & Fairbain 2007). Finally, when are the molecular details underlying trade-offs important and when can they be ignored when predicting diversity at ecological scales? Partial in-roads have been made in addressing these issues, as explained in examples described in this study and in examples elsewhere (Knight *et al.* 2006; Bennett & Lenski 2007; Jasmin & Kassen 2007).

Moving forward, we propose that there are general principles that may be applied broadly in efforts to understand the microscopic basis for trade-offs and the ecological implication of specific trade-off shapes for patterns of diversity. One goal of future research, from modelling and experimental perspectives, is to treat functional traits as deriving from measurable subcellular parameters. The ecological relevance of ‘predicting numbers from alphabets’, or moving from sequences to quantitative traits and trait trade-offs (Kim *et al.* 2009), will require the inclusion of community structure to evaluate how important it is to quantify parameters within microbial gene regulation and biophysical interactions on an ecological scale. These parameters would include kinetic constants related to gene regulation, such as binding rates and transcriptional rates, as well as biophysical constants, such as activation energy of bonds related to structure and stability. In developing eco-evolutionary models and experimental assays, bacterial or viral mutants should be viewed as differing in terms of measurable molecular parameters, which map onto life history traits via models of biophysics and quantitative gene regulation. Then, predictions of the success of mutants would depend on the relative fitness at the ecological scale. This link between studies of intracellular functioning, trait space models and eco-evolutionary dynamics will be worth the investment of time, resources and interdisciplinary conversations if models can guide the unexpected discovery of trade-offs and/or the explanation for why microbes have such a massive range of functional traits. The danger may be that in doing so, more realistic models may, instead, lead to increases in precision but decreases in generality and scope of applicability (e.g. Levins 1966).

Obviously, closing the loop between molecular mechanisms underlying trade-offs and predictions regarding the outcome of evolution remains a challenge. Part of this difficulty is because as much as we may want to implement general rules for relatively simple systems and then use those rules to predict what should happen in complex communities, experiments suggest that biophysical details and ecological interactions often matter – details which we sometimes cannot know in advance and which may simply be idiosyncratic. Mathematical models that are specific enough to allow for quantitative predictions about the microbial system at hand, yet general enough to allow for qualitative predictions to be made beyond the constraints of the specific experimental system can provide insights into when these details matter (Forde *et al.* 2008; Gavrilets & Losos 2009). For example, by considering a case study of the relationship between host–pathogen diversity and resource availability, Forde *et al.* (2008) used an approach that addressed the following question: How can we determine whether an experimental result is particular to the *in vitro* model, and if so, can we characterize the systems likely to behave differently and understand why? A mathematical model containing sufficient sub-cellular detail so that it quantitatively captured experimental results of a given study system was developed. Then by searching through parameter space, a classification of all possible dynamics was made to determine whether a given experimental outcome held across different microbial systems and different environments or whether it was system-specific. Thus, a combination of *in vitro* systems and appropriately configured mathematical models can be an effective means for tackling the above issues of generalizability.

It is also possible that trade-offs of ecologically important traits may not exist. For example, a trade-off might not be observable when there is a de-coupling of metabolic and/or regulatory pathways associated with traits of interest. In addition, a trade-off might not be directly observable when mechanistic manipulations of microbes are unavailable. In such cases, an ecological trade-off could still be mapped to statistical correlations among the expression of genes or gene pathways using next-generation sequencing technologies. Such an outcome would be analogous to the use of correlative approaches to analyse trait trade-offs, albeit at a different scale. For example, the existence of statistical trade-offs between the expression of genes that underlie metabolic capacity, stress resistance and viral infection would be invaluable in gaining some insight as to the functional diversity and community structure of expected microbes within an environment. Statistical trade-offs of this kind will be of relevance to environmental microbiologists interested in the potential for microbes to sequester an environmental pollutant and to ecologists interested in patterns of diversity at the microbial scale. In the long term, discovering intracellular trade-offs will stimulate hypotheses regarding the mechanistic basis of trade-offs. A similar approach is being utilized at the intersection of medical microbiology and ecology and has already yielded noteworthy successes (Ley *et al.* 2006; Klepac-Ceraj *et al.* 2010).

Despite these cautions, the rewards of the approach we espouse may be worth the effort because forging a multi-scale approach to diversity has the possibility of shedding light on macro-ecological patterns whose explanations reside, at least partially, in intracellular details. One such example is that of Klausmeier *et al.* (2004), whose aim was to understand how intracellular trade-offs can explain the nearly ubiquitous finding that ratios of nitrogen to phosphorous (N : P) in deep oceans is *c.*16 : 1, also known as the Redfield ratio (Redfield 1958). The cellular machinery required for phytoplankton to grow quickly (ribosomal RNA) has a lower nitrogen to phosphorous ratio than cellular machinery used for the uptake of nutrients (transporter proteins on the cell membrane). Using a combination of differential equations for nutrient and light usage, parameterized with kinetic and physiological constants from laboratory experiments, Klausmeier *et al.* (2004) predicted which species (those that have high or low N : P ratios) would dominate different environments. Whether phytoplankton are N or P limited selects for distinct optimal allocations of intracellular machinery. The allocation of intracellular machinery corresponds to distinct N : P whole cell ratios, bracketing the finding of the Redfield ratio. This promising result leaves unresolved the questions of why the Redfield ratio emerges and why so many phytoplankton coexist, questions that will likely require the continued combination of molecular and ecological perspectives.

The challenge we have identified in this paper is to connect the intracellular details underlying ecologically relevant trade-offs with the dynamic processes of diversification among microbes. This challenge is not only feasible, but has parallels in macro-organisms. Indeed, the field of ‘evo-devo’ and its studies of the diversity of eukaryotic development have validated the importance of searching in the intracellular realm to understand the constraints on macroscopic form and the ensuing ‘endless forms most beautiful’ (*sensu*Darwin 1859; Carroll 2005). As the field of ecological genomics grows, we are learning more and more about the vast diversity of microbes across ecosystems. However, numerous questions about the ecological and physiological relevance of this sequence diversity are arising as a result. The combined use of mathematical modelling, laboratory experiments and ecological genomics will provide a unique opportunity to understand both the intracellular and ecological mechanisms that drive microbial diversity in nature.
